# Impact of a Physician Clinical Support Supervisor in Supporting Patients and Families, Staff, and the Health-Care System During the COVID-19 Pandemic

**DOI:** 10.1017/dmp.2020.345

**Published:** 2020-09-10

**Authors:** Sarah E. Tevis, Hemali Patel, Sarguni Singh, Cathy Ehrenfeucht, Charlie Little, Jean Kutner, Jason Persoff

**Affiliations:** 1 Assistant Professor of Surgery, Department of Surgery, University of Colorado School of Medicine, Aurora, Colorado; 2 Associate Professor of Medicine, Division of Hospital Medicine, Department of Medicine, University of Colorado School of Medicine, Aurora, Colorado; 3 Vice President Director of Operations and Associate Chief Nursing Officer, University of Colorado Hospital/UCHealth, Aurora, Colorado; 4 Associate Professor of Emergency Medicine, Medical Director for Emergency Preparedness, Department of Emergency Medicine, University of Colorado School of Medicine, Aurora, Colorado; 5 Professor of Medicine, Division of General Internal Medicine, Department of Medicine, University of Colorado School of Medicine, Chief Medical Officer, University of Colorado Hospital, Aurora, Colorado; 6 Associate Professor of Medicine, Assistant Medical Director of Emergency Preparedness, Division of Hospital Medicine, Department of Medicine, University of Colorado School of Medicine, Aurora, Colorado

**Keywords:** health services, leadership, delivery of health care

## Abstract

As coronavirus disease 2019 (COVID-19), caused by severe acute respiratory syndrome coronavirus 2 (SARS-COV2), became a pandemic, hospitals activated Hospital Incident Command Systems (HICS). At our institution, we included a role of Physician Clinical Support Supervisor (PCSS) in the HICS structure. The PCSS role was filled by physicians who served hospital leadership positions, such as Physician Advisor, Medical Staff leadership, Chief Quality Officer, and Chief Medical Informatics Officer. In an effort to summarize the lessons learned by implementation of the PCSS role during the COVID-19 pandemic, we evaluated a PCSS working Microsoft Teams™ spreadsheet and the experience of physicians in the PCSS role. Through efficient daily 2-way communication between frontline providers, HICS, and hospital leadership, the PCSS role facilitated rapid change and improved support for frontline staff, patients and families, and the health-care system. We recommend including the role of PCSS in HICS structure in the event of future pandemics or other crises.

The novel coronavirus disease 2019 (COVID-19), cause by severe acute respiratory syndrome coronavirus 2 (SARS-COV2), was first described in Wuhan City, China in December, 2019, and rapidly spread worldwide reaching a pandemic level in a matter of months.^1^ During a pandemic, hospitals face many challenges including caring for a growing number of critically ill patients; building rapid, nimble care processes; implementing testing protocols; expanding facilities; maintaining adequate personal protective equipment (PPE) and supplies; and addressing financial concerns.^2^ Hospitals across the country activated Hospital Incident Command Systems (HICS) to provide an organized, all-hazards approach to the myriad of challenges that arose on a daily basis.

At our institution, we developed a HICS role called the Physician Clinical Support Supervisor (PCSS), staffed by physicians in hospital leadership positions.^3^ The PCSS met daily with the primary clinicians caring for patients with COVID-19, including critical care and hospital medicine COVID-19 teams, serving as a point of contact for physicians and frontline staff to answer COVID-19-related questions and identify opportunities to improve care delivery to hospitalized patients. To track arising issues and monitor progress on solutions, the physicians filling the PCSS role used a working Microsoft Teams™ spreadsheet documenting frontline providers’ problems and concerns, as well documenting identified solutions once issues were resolved. This document served as closed loop communication to allow all physicians filling the role of PCCS to keep up to date on the latest information. This allowed for effective, timely communication of relevant information to the COVID teams in the setting of rapidly changing information, while also keeping other hospital leadership abreast of the issues and progress.

The PCCS role allowed fluid communication between HICS leadership and frontline providers and facilitated identification of barriers and solutions to providing high quality care to both COVID-19 and non-COVID-19 patients in this unprecedented time. Our aim was to use the PCSS working Microsoft Teams™ spreadsheet and the experience of physicians in the PCSS role to outline lessons learned during the initial influx and peak of COVID-19 admissions over a 2-mo period at our institution. We summarize the lessons learned into the following categories: supporting frontline staff, supporting patients and families, and supporting the health-care system (Figure 1).

**FIGURE 1:**
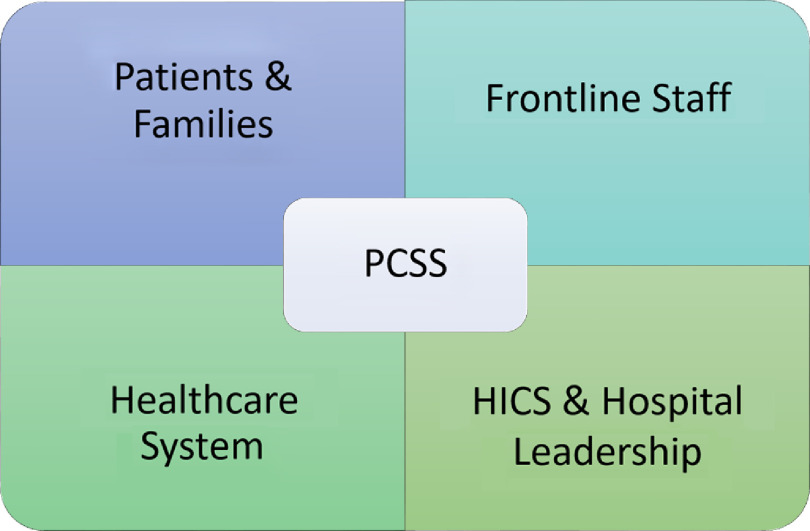
Role of the PCSS in Bidirectional Communication. The PCSS played an essential role in bidirectional communication between frontline staff and HICS and hospital leadership, thereby supporting patients and families, frontline staff, and the health-care system.

## SUPPORTING FRONTLINE STAFF

### Bidirectional Communication and Education

The PCSS role was integral in supporting HICS and frontline staff communication and education needs. Early in the COVID-19 pandemic, the PCSS worked closely with HICS leadership and infection control to develop protocols for COVID-19 isolation, testing, and patient management. The PCSS role then became fundamental in providing education and improving adherence to policies at a time when staff were overwhelmed with information overload in the form of multiple daily emails; rapidly changing information and evidence; and information from outside sources, such as the media, social media, and family and friends. The PCSS worked daily to address frontline staff concerns regarding PPE, testing, high-risk exposure, and quarantine/isolation policies for exposed staff. The unique 1-on-1 capability of the PCSS to field questions, review updated hospital guidelines, and attend daily HICS meetings to learn about potential upcoming changes proved invaluable to frontline staff. The PCSS was also available to facilitate conversations and mediate conflict when debate ensued about COVID-19 testing or type of isolation for any given patient.

### Development of Efficient Processes of Care

By identifying concerns and inefficiencies from the frontlines (Table 1), the PCSS role was able to proactively communicate these issues to hospital leadership and rapidly develop new processes to streamline care, making it easier for frontline staff to care for their patients. Some examples of this include: (1) creating a specific home oxygen pathway for COVID-19 patients due to their high oxygen needs with exertion; (2) COVID-19 testing pathways for patients discharging to off-site facilities; and (3) testing pathways for transplant recipients and immunocompromised patients.

**TABLE 1:** Role of the PCSS in Identifying Issues and Solutions

Issue	Group Affected	PCSS Action	Result
Concern about discharging patients with high oxygen requirements with exertion	Patients with oxygen levels >2 L/minwith exertion	Group coordination to develop a protocol involving multiple groups	New Home Oxygen Pathway with specific attention to this group of patients
Patients unable to discharge to care facilities due to COVID-19	Patients no longer meeting inpatient criteria but unable to return home	Group coordination to determine what care facilities would deem acceptable acceptance criteria	Negative COVID-19 nasopharyngeal swabs times two was considered acceptable risk and patients then were transferred successfully to care facilities
Lack of standardization in discharge care for patients with COVID-19	All COVID-19-positive inpatients	Group coordination between extensive stakeholders including outpatient providers, telehealth monitoring, and respiratory therapy	Standardized discharge pathway easily orderable through the EMR with the additional benefit of standardized “dot phrases” allowing standardized verbiage in the chart and for home-going
Respiratory therapy overwhelmed by need for home oxygen to be completed coupled with increase in tracheostomy care and complex respiratory issues	Respiratory therapy	Group creation of a new group of back-up respiratory therapists	Allows main respiratory therapists to provide direct patient care while back-up therapists completed the challenging home oxygen process

Probably the most effective example of the PCSS role was contributing to the creation and standardization of the discharge process for COVID-19 patients. This included the development of new standardized phrases in the electronic medical record (EMR) to standardize communication to patients, discharge and follow-up pathways, and discharge planning for complex patients with new tracheostomies. Many patients required home oxygen and that process was made more efficient by linking the home oxygen order and home oxygen evaluation orders in the EMR, creating a system of backup respiratory therapists (RTs) so that RT staffing could be flexed up as needed, and offloading RTs by relieving them of other duties such as albuterol administration.

### Bolstering a Culture of Safety

Frontline staff safety in caring for patients with COVID-19 was paramount and a major area of focus for HICS and the PCSS role. Many practical considerations raised by frontline staff were addressed by the PCSS in real time. For example, as more patients were tested for COVID-19 and became people under investigation (PUIs), the PPE process required streamlining to prevent unnecessary exposures and conservation of PPE. Examples included regular staff and processes to build and maintain isolation carts in dedicated locations on COVID-19 units and in the emergency department (ED). Furthermore, many patient rooms did not have disposable stethoscopes requiring staff to leave the room to obtain a stethoscope thereby using more PPE and wasting provider time rather than focusing on patient care. Protocols were established to ensure a standard location for hanging the stethoscopes in patient rooms and to ensure stethoscopes would travel with patients from the emergency room to their hospital room or between units once admitted to the hospital. Finally, protocols were established for provider and staff safety, especially when transitioning home to minimize any exposure to their family. This included shower access at the on-campus exercise facility 7 d per wk from the hours of 6:00 am to 12:00 am so staff could shower after their shift. Scrub deposit was made available at the showers so staff could easily return soiled scrubs. Additional call rooms were also opened for the added staff working in the intensive care unit (ICU) at night.

### Advance Care Planning

It was clear in discussions with the teams caring for COVID-19 patients that frontline providers had significant concerns about the high mortality and end of life care COVID-19 patients might require when hospitalized. As an institution, we made it a priority to determine patients’ wishes at the time of admission. Hospital medicine physicians on the COVID units suggested creating advance care planning packets that included a Medical Durable Power of Attorney (MDPOA) form as well as a Medical Orders for Scope of Treatment (MOST) form. Mandatory completion of the advance care planning packet at the time of admission facilitated minimizing the use of PPE, optimizing the use of interpreters, and ensuring patients’ wishes were identified and documented accurately. Standardized phrases and EMR reminders were also created to facilitate addressing code status and providing links to both English and Spanish MDPOA forms. In addition, a process was developed to upload the signed MDPOA forms into the EMR without transferring potentially contaminated paperwork outside of the patients’ rooms. Finally, we built a new care management process to increase completion of MDPOA paperwork from 25.8% (pre-COVID-19) to 40.2% (during COVID-19).

The PCSS also collaborated with multiple care teams to develop prompts and criteria for early palliative care consults for all hospitalized patients with COVID-19. In addition, the palliative care teams were physically located in the ICU and the ED conducting daily outreach to the Hospital Medicine teams to try to see high-risk patients early during their hospitalization. The chief medical officer (CMO) frequently advised on discussions where providers determined that a patient was receiving nonbeneficial interventions, eventually having the PCSS participate in ensuring backup for the frontline providers in enacting often challenging discussions and decision-making. This created a streamlined process for providers to receive assistance in these challenging scenarios.

We also changed our CODE blue process to ensure staff had adequate PPE, to minimize the number of people in the room, and to preserve PPE. The size of the CODE blue teams was decreased from 16 members to 12 members with only 8 team members entered patient rooms during cardiac arrests. Each member of the CODE blue teams received a fanny pack for their individual PPE. PPE Crash Packs were created and included 2 N95 masks (1 medium, 1 large), 1 gown, 4 pairs of gloves (2 medium, 2 large), and 2 face goggles. Rapid sequence intubation medications were added to COVID-19 units and a process was developed for instances where more than 1 CODE was called simultaneously.

## SUPPORTING PATIENTS AND FAMILIES

### Unmet Patient Needs

Expanding isolation precautions, preserving PPE for patients, and protecting staff from exposure to COVID-19 created unique challenges to providing the usual efficient and effective standards of care. There was a disproportionate volume of non-English speaking patients admitted during the initial surge of inpatient COVID-19 patients. To improve communication, we provided translation devices in rooms for non-English speaking patients to facilitate communication while preserving PPE. We also provided patients with iPads to interact with family members with those devices dedicated to a specific patient room to reduce cleaning and PPE use. In addition, we preferentially placed patients with COVID-19 in rooms with windows to the hallway, thereby allowing providers to call into the room to talk to the patient, but still maintain interpersonal distance. Patient care carts were created to provide patients with activities as they had less face-to-face interactions.

Many of the challenges identified by the PCSS revolved around building safe, effective transitions of care processes, such as arranging home oxygen, ensuring safe follow-up after discharge, and addressing delays in placing patients who required a rehabilitation or nursing facility after discharge. Two home oxygen coordinators facilitated discharging patients on oxygen, performing a pilot study using the Masimo™ device to monitor oxygenation after discharge for high-risk patients; postdischarge phone calls ensured patients received adequate follow-up. Leveraging the electronic patient portal (MyHealthConnection™), an app allowing individuals to access their own medical record, while they were in the hospital facilitated the use of virtual health visits after discharge. For uninsured patients or underinsured patients without a primary care provider, we built a process where a nurse would call the patient on discharge to ensure the transition home was safe if we were unable to establish follow-up care with a clinic. Many discharge delays were secondary to lack of placement options for COVID-19 patients; therefore, physical therapy began triaging all COVID-19 patients and appropriately deploying resources to provide more intensive services for patients similar to the services they would receive if they were discharged to a nursing or rehab facility. Furthermore, physiatrists opened a rehabilitation unit within the hospital to transition COVID-19 patients to a service who required ongoing but few other medical needs to offload medical services so they could care for patients who required hospitalization.

### Family and Visitors

Another challenge for patients, as well as family and visitors, included the hospital-wide no visitor policy. This lack of family support placed undue burden on patients, but was necessary to protect families, patients, staff, and physicians. As previously noted, we were able to provide patients with iPads so they could video chat with friends and family daily. There was also a process for exceptions to the no-visitor policy in cases such as labor and delivery, patients who were minors, and end of life. These exceptions were posted on the hospital intranet, all exceptions were approved by HICS, and exceptions were communicated to hospital security by HICS.

Communication with families also proved challenging with the visitor policy. Through discussions between providers and the PCSS, we identified that multiple family members and friends were calling the nursing stations daily for updates, and it became a struggle for nurses and physicians to keep up with the many requests for information. To address this challenge, a communication nurse was enlisted on each unit and was responsible for fielding family member calls and updating families daily. Expectations were also added to the communication provided to patients and families at the time of admission, outlining the importance of identifying 1 family member to communicate with the medical team who could relay information to other family and friends.

## SUPPORTING THE HEALTH-CARE SYSTEM

In addition to providing education to frontline staff from HICS, the PCSS acted as a conduit of information from frontline staff to HICS and hospital leadership. By interacting daily with the teams caring for COVID-19 patients, the PCSS was able to closely monitor frontline staff, thus informing the system and advocating for concerns to be addressed in real time. In addition, the PCSS often was able to quash rumors and misinformation through having a lifeline to these provider teams. Gathering of this qualitative data daily provided valuable information at an organizational level that was used to address staff concerns and questions before frustrations mounted. For example, many of the topics discussed at system-wide Town Hall meetings were brought to the PCSS by frontline staff including concerns about asymptomatic patients, staff becoming ill, and larger capacity management plans.

Conflict can occur if there is ineffective communication between hospital leadership and frontline providers, especially during these stressful times of rapid change. This can lead to frustration, delays in care, and possible unsafe patient care. The PCSS can play an integral role as liaison between hospital leadership and frontline providers. The PCSS understands the requirements in providing frontline care and can successfully foster a partnership with frontline clinicians and staff. At the same time, they understand the challenges of building and maintaining hospital level processes as well as leadership challenges on the hospital side. This role can, therefore, bridge the gap that is often present between administration and clinical frontline providers. During these unprecedented times, this role is particularly necessary to foster communication to provide safe, effective care.

## CONCLUSIONS

The novel PCSS position in the HICS provided efficient 2-way dialog between frontline staff and HICS and hospital leadership. The PCSS was able to facilitate numerous changes to better support staff and uncover and address unmet patient needs in real time, while supporting HICS and hospital leadership. We recommend a dedicated physician role in HICS structure in the event of future pandemics or other crises.

## References

[1] Rothan HA , Byrareddy SN. The epidemiology and pathogenesis of coronavirus disease (COVID-19) outbreak. J Autoimmun. 2020;109:102433. 32113704 10.1016/j.jaut.2020.102433 PMC7127067

[2] US Department of Health and Human Services. Hospital experiences responding to the COVID-19 pandemic: Results of a national pulse survey. PS Net Website. https://psnet.ahrq.gov/issue/hospital-experiences-responding-covid-19-pandemic-results-national-pulse-survey-march-23-27. Published April 3, 2020. Accessed June 20, 2020.

[3] Persoff J , Patel H , Singh S , et al. Expanding the hospital incident command system with a physician-centric role during a pandemic: the role of the physician clinical support supervisor. J Hosp Adm. 2020. doi: 10.5430/jha.v9n3p7

